# Adaptive gait responses to varying weight‐bearing conditions: Inferences from gait dynamics and H‐reflex magnitude

**DOI:** 10.1113/EP091492

**Published:** 2024-03-15

**Authors:** Yong Kuk Kim, Michelle Gwerder, William R. Taylor, Heiner Baur, Navrag B. Singh

**Affiliations:** ^1^ Laboratory for Movement Biomechanics, Institute for Biomechanics, Department of Health Sciences and Technology ETH Zurich Zurich Switzerland; ^2^ Department of Biomedical Engineering University of Basel Basel Switzerland; ^3^ School of Health Professions, Physiotherapy University of Applied Sciences Bern Switzerland; ^4^ Singapore‐ETH Centre, Future Health Technologies Program Singapore Singapore

**Keywords:** balance, H‐reflex, loading conditions, spinal reflex, walking

## Abstract

This study investigates the effects of varying loading conditions on excitability in neural pathways and gait dynamics. We focussed on evaluating the magnitude of the Hoffman reflex (H‐reflex), a neurophysiological measure representing the capability to activate motor neurons and the timing and placement of the foot during walking. We hypothesized that weight manipulation would alter H‐reflex magnitude, footfall and lower body kinematics. Twenty healthy participants were recruited and subjected to various weight‐loading conditions. The H‐reflex, evoked by stimulating the tibial nerve, was assessed from the dominant leg during walking. Gait was evaluated under five conditions: body weight, 20% and 40% additional body weight, and 20% and 40% reduced body weight (via a harness). Participants walked barefoot on a treadmill under each condition, and the timing of electrical stimulation was set during the stance phase shortly after the heel strike. Results show that different weight‐loading conditions significantly impact the timing and placement of the foot and gait stability. Weight reduction led to a 25% decrease in double limb support time and an 11% narrowing of step width, while weight addition resulted in an increase of 9% in step width compared to body weight condition. Furthermore, swing time variability was higher for both the extreme weight conditions, while the H‐reflex reduced to about 45% between the extreme conditions. Finally, the H‐reflex showed significant main effects on variability of both stance and swing phases, indicating that muscle‐motor excitability might serve as feedback for enhanced regulation of gait dynamics under challenging conditions.

## Abstract (highlights)

- **What is the central question of this study?**
  How do varying weight‐loading conditions influence neural excitability and gait dynamics, particularly focusing on the H‐reflex and foot placement during walking?
- **What is the main finding and its importance?**
  Different weight conditions significantly impacted foot timing and gait stability. With weight reduction, there was a notable decrease in double limb support time, step width and H‐reflex. The result suggested that weight reduction posed more challenges than weight addition. This has implications for understanding neural responses during varied weight‐loading scenarios.

## 1 INTRODUCTION

Walking is the most common daily activity, seemingly uncomplicated, but involving intricate control processes involving the cortex, brain stem and spinal cord (Al‐Yahya et al., 2019; Meester et al., 2014). The human sensorimotor system continually integrates proprioceptive, vestibular and visual information to regulate the stepping behaviour and maintain the centre of mass (CoM) within the base of support (BoS). For those reasons, kinematics and inter‐stride variations in timing and placement of the feet during walking have been rigorously exploited to understand behavioural adaptation (Callisaya et al., 2011; Hausdorff, 2005; Hausdorff et al., 1998, 2001; Hollman et al., 2016; König Ignasiak et al., 2019; Konig, Singh, et al., 2016; Konig, Taylor, et al., 2016; Menz, 2003; Owings & Grabiner, 2004; Ravi et al., 2020). Reductions in mean gait parameters, such as speed and stride length, indicate a ‘cautious’ gait, often seen in Parkinson's disease (Konig, Singh, et al., 2016; Pistacchi, 2017; Ravi et al., 2020). Moreover, greater foot placement and timing variability suggest instability responses (König Ignasiak et al., 2019; Sivakumaran et al., 2018), linked to higher fall risk (Ambrose et al., 2013; Callisaya et al., 2011; Cameron & Nilsagard, 2018; Hausdorff et al., 2001; Mitra & Fraizer, 2004; Pekny et al., 2015), and increases with task demands (Filli et al., 2019; Hausdorff et al., 1998, 2001). A better understanding of how healthy individuals control their walking under different challenges (various weight‐loading conditions) could lead to new therapeutic strategies.

The short latency Hoffman reflex (H‐reflex) is extensively used to assess the excitatory behaviour of the monosynaptic Ia‐afferent volleys in the spinal cord circuitry, providing insights into the neural mechanisms underlying locomotion (Knikou, 2008; Palmieri et al., 2004; Schieppati, 1987). For instance, healthy individuals experience a decreased H‐reflex when met with unfamiliar tasks (increasing task demands), reflecting more cortical control (Capaday & Stein, 1987; Johannsson et al., 2019; Kaneko et al., 2018; Zehr, 2002). Hence, down conditioning of the H‐reflex is used as an effective rehabilitative strategy plan for individuals with a spinal cord injury (Kaneko et al., 2018; Thompson & Wolpaw, 2021). Varying weight‐bearing conditions are a common experience that people face in a daily manner (e.g., carrying groceries or wearing a backpack), inducing altered gait and posture as they influence muscle activation and gait (Barela et al., 2019; König Ignasiak et al., 2019), affecting H‐reflex amplitude and patterns (Ferris et al., 2001).

While much is known about the independent operation of the central and peripheral nervous systems (Al‐Yahya et al., 2019; König Ignasiak et al., 2017; Luu et al., 2012; Phadke et al., 2006; Tsuruike et al., 2012), little is known about how these mechanisms interact when a person is walking. Specifically, there is still a gap in our understanding of the role of descending motor pathways regulating motor output during walking. This gap is especially pronounced when we unravel the adaptive responses necessary for the varied and unpredictable walking tasks prevalent in modern environments. For example, while studies have investigated the role of underlying motor control mechanisms as well as the changes in gait under varying load conditions (Ferris et al., 2001; Phadke et al., 2006; Phadke et al., 2007; Tsuruike et al., 2012), what is relatively unknown is how individuals adapt to mechanical demands during walking and the associated and crucial excitatory or inhibitory inputs to motor neurons.

The aim of our study was therefore to investigate gait adaptations as well as the associated excitatory status of the soleus muscle, via the H‐reflex, under diverse mechanical loads. We introduced a range of weight conditions that were both familiar, for example, additional weight using a backpack commonly encountered in everyday life, and unfamiliar, for example, reduced weight or a harness‐based body weight support system. The idea was to gain a deeper understanding of gait adaptations as well as the H‐reflex amplitude of the soleus muscle.

## 2 METHODS

### 2.1 Ethical approval

The study complied with the *Declaration of Helsinki*, except for registration in a database: each subject provided written informed consent prior to participation (BASEC‐No. 2019‐00678), which was approved by the Kantonale Ethikkommission Zurich, Switzerland. All measurements were performed in the Laboratory for Movement Biomechanics at the Institute for Biomechanics at ETH Zurich.

### 2.2 Participants

Twenty healthy participants (11 males and nine females, with mean ± standard deviation (SD): 24.20 ± 2.12 years; 174.35 ± 7.54 cm; 69.34 ± 8.24 kg) were recruited from the local community. None of the participants reported any history of musculoskeletal diseases, neurological disorders, acute pain or artificial joints. Consumption of alcohol and heavy physical activity were avoided 24–48 h prior to the experiment.

### 2.3 Experimental design, procedures and protocols

After signing the informed consent, participants were equipped with a lower body set of 31 reflective markers captured using a 3D optical motion capture system (Vicon Motion Systems Ltd, Oxford, UK), with at least four markers on each body segment. Subsequently, participants had surface electromyography (sEMG) electrodes (Delsys Inc., Natick, MA, USA) fixed according to the SENIAM guidelines on the following muscles of both legs: tibialis anterior (TA), gastrocnemius lateralis (GL), gastrocnemius medialis (GM), vastus medialis (VM), vastus lateralis (VL), semitendinosus (SM) and soleus (SOL). Following placement of electrodes for eliciting H‐reflex response (details below), participants familiarized themselves with walking on the treadmill (Pulsar 2 Generation, h/p/cosmos Sports & MedicalGmbH, Nussdorf‐Traunstein, Germany). The sampling frequency of sEMG was 3000 Hz. They were then requested to walk at their preferred walking speed (mean ± SD overall walking speed was 3.28 ± 0.41 km/h) to elicit the H‐reflex recruitment curve. This recruitment curve was then used for standardizing H‐reflex responses.

This study then investigated the following five conditions in a randomized order: (1) body weight walking (Normg), (2) 20% additional body weight (Wei_20_) with an additional weight provided using a upper‐body weight vest, (3) 40% additional body weight (Wei_40_), (4) 20% reduced body weight (Har_20_) where reduction was achieved using body weight support harness system (MasterVest, Liko, Sweden), and (5) 40% reduced body weight (Har_40_). Bodyweight reduction was confirmed through a strain gauge assessment built within the harness system. Finally, participants walked barefoot on the treadmill for 6 min under each condition during which the H‐reflex was assessed in the dominant leg, as described below. The dominant leg was determined by asking, ‘If you would shoot a ball towards a target, which leg would you use to shoot the ball?’ (van Melick et al., 2017).

#### 2.3.1 H‐reflex

##### Placement of the electrodes

The anode (40 × 90 mm, Spes Medica, Genova, Italy) of the H‐reflex stimulation electrode was placed 2 cm above the top of the patella on the dominant leg (Ozyurt et al., 2018). To determine the location of the tibial nerve for the cathode placement, a line was marked across the length of the popliteal fossa's crease at 20° knee flexion while the participant lay in a prone position. The tibial nerve is known to be located between the midpoint of this line and a second point 10–15 mm laterally of the midpoint (Ozyurt et al., 2018). The cathode (Ag/AgCl Sintered Electrodes—Extra Flat, Neurospec, Stans, Switzerland) was then firmly pressed between these two points and fixed using adhesive tape to prevent relative movement.

##### Evoking H‐reflex response

**FIGURE 1:**
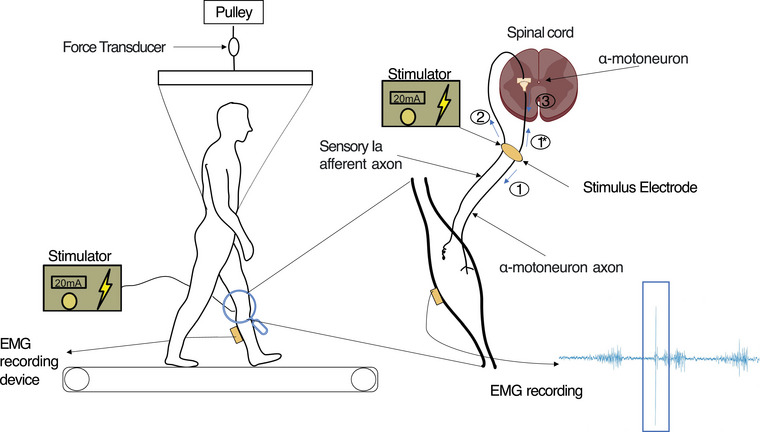
Low‐level electrical stimulation triggers the H‐wave (the secondary EMG wave in Figure 2b), while higher intensity increases the M‐wave (the primary wave in Figure 2b). The Ia sensory neuron is comapratively larger than the motor axon, making it easier to excite (response 2). This sensory input induces an action potential on the α‐motoneuron axons that propagates to the target muscle (response 3), resulting in the H‐reflex wave. Suppose the stimulation is over the threshold of the α‐motoneuron axons: it creates a potential right at the stimulation point that can go in two directions – to the muscle (producing the M‐wave, response 1) and back to the spinal cord (response 1*). If these two potentials intersect (response 3), the H‐wave can be reduced or even nullified.

The SOL H‐reflex was evoked by stimulating the tibial nerve (rectangular pulse, 1 ms duration) with an electrical stimulator (DS7A, Digitimer Ltd, Welwyn Garden City, UK), for which the timing of electrical stimulation was controlled using a custom script programmed in MATLAB (2018a, The MathWorks Inc., Natick, MA, USA). Elicitation of an H‐reflex response using this set‐up was confirmed in a standing position with the SOL sEMG sensor (Figure 1a). EMG recording parameters were set with temporal windows to identify the M‐wave and H‐reflex responses. The M‐wave was expected to be between 5 and 15 ms, and the H‐reflex was expected to be between 30 and 45 ms post‐stimulus. Four parameters were evaluated as peak‐to‐peak (p‐p) amplitude from the SOL sEMG signal: (a) background sEMG during swing phase (bEMG_swing_), (b) background sEMG obtained after the heel strike (bEMG_stance_) but prior to the corresponding heel strike stimulus (Knikou, 2008), (c) M‐wave signal directly travelling along the efferent fibres after stimulation, and (d) H‐reflex, both M‐Wave and H‐reflex are obtained from sEMG after heel strike (Figure 1b,c). The peak‐to‐peak amplitude, as opposed to rectification and averaging, offers a more accurate reflection of the neuromuscular system's state and is less susceptible to signal artefacts and baseline noise (Dideriksen & Farina, 2019; Neto & Christou, 2010)

##### Recruitment curve

**FIGURE 2:**
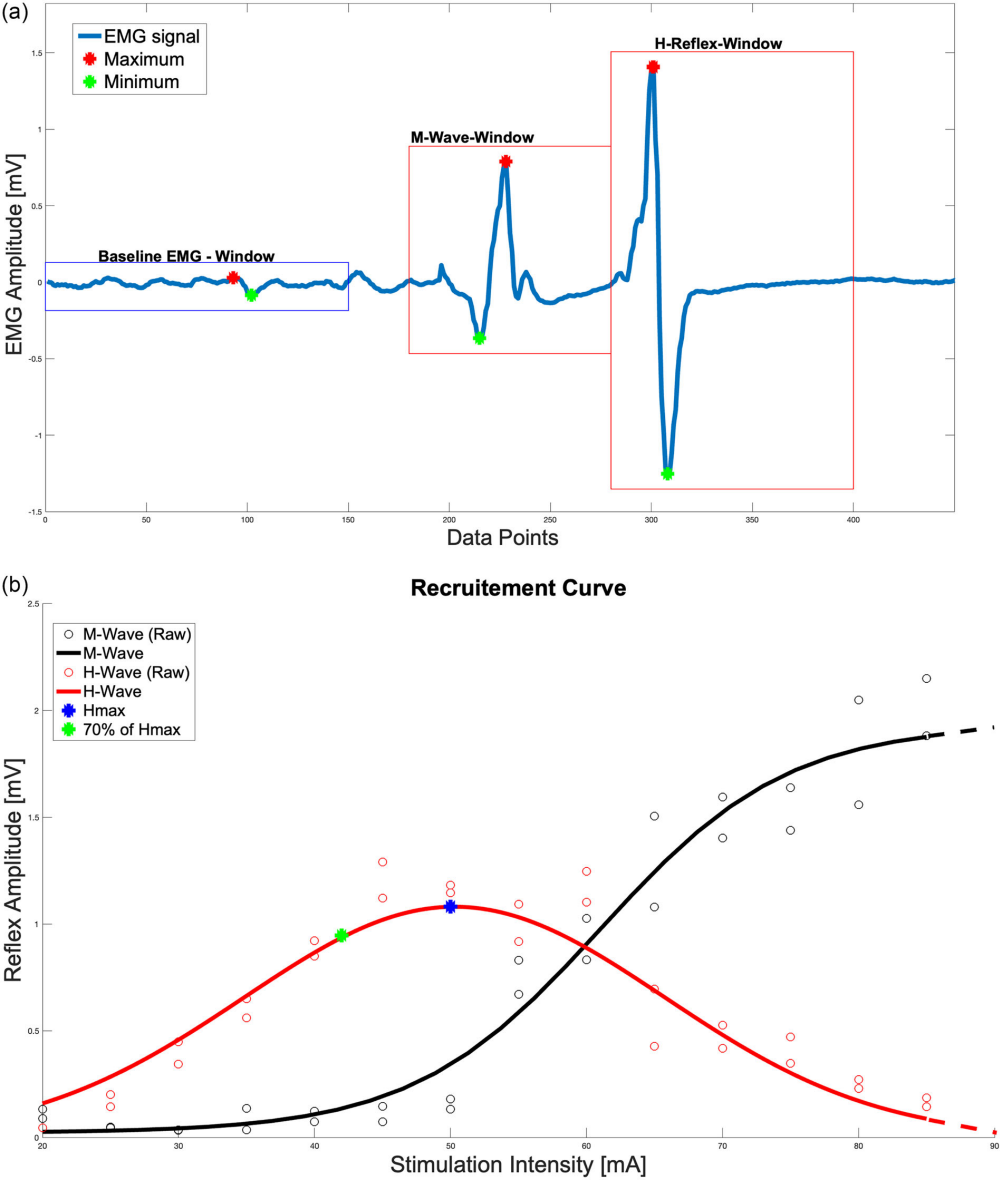
H‐reflex and M‐wave. (a) Filtered EMG signal of bEMG, M‐wave and H‐reflex, highlighted with red box from left to right, respectively. (b) An example of a recruitment curve: with increasing stimulus intensity the H‐reflex starts to increase and later on the M‐wave starts to increase.

**FIGURE 3:**
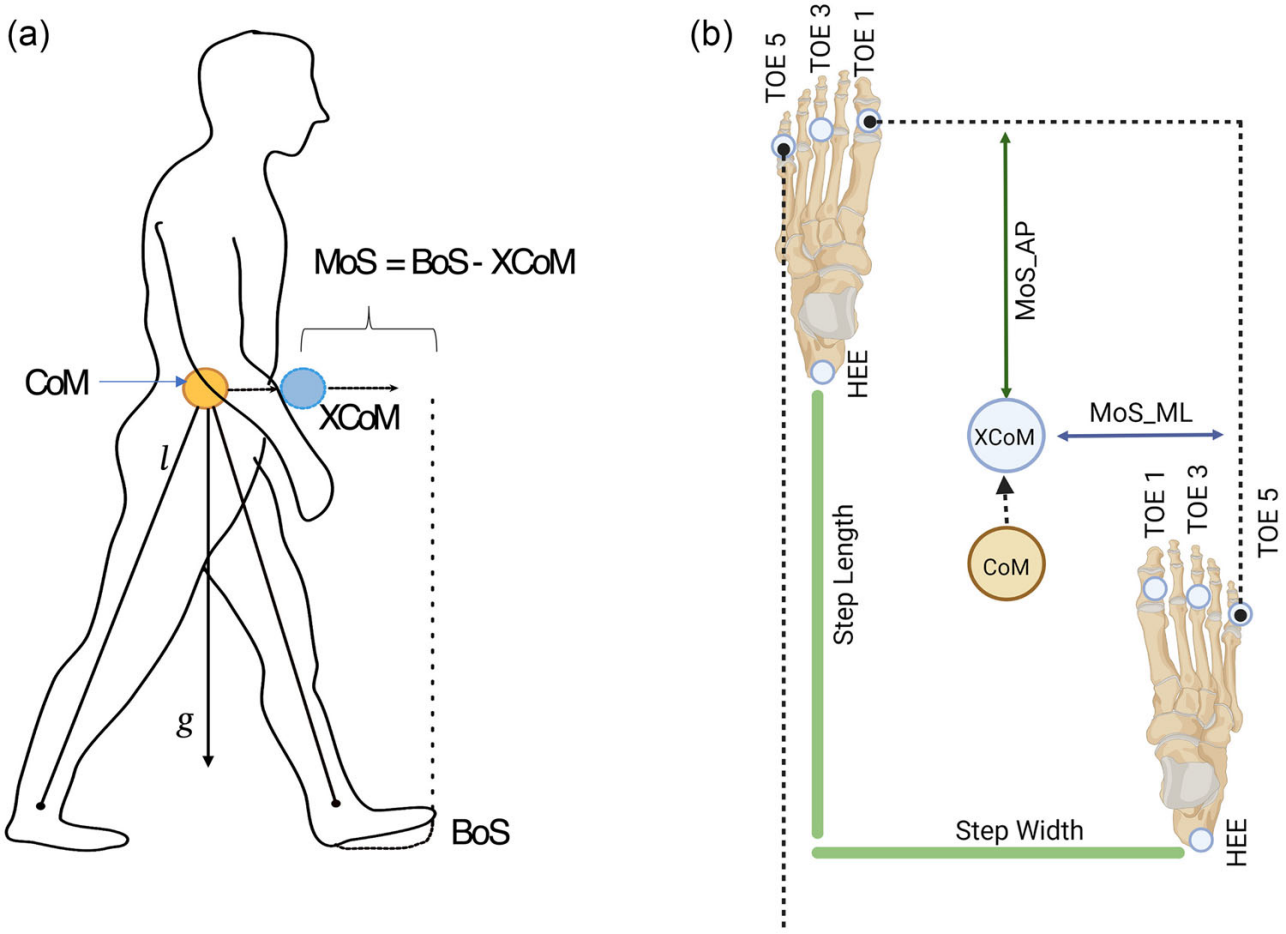
(a) Inverted pendulum model with pendulum length 𝑙, centre of mass (CoM), gravitational acceleration 𝑔 and base of support (BoS). (b) The position of the extrapolated centre of mass (XCoM) and the frontal and lateral borders of the BoS are defined.

The stimulus intensity to obtain the recruitment curve was increased at increments of 5 mA starting at 20 mA for all participants (Figure 2b). Each participant was stimulated twice at every intensity to ensure consistency, and the stimulations occurred shortly after the heel strike. The motion capture system was used to precisely detect the onset of heel strike events by tracking the position of the markers on the feet (Figure 3). Once identified, the stimulus was triggered using the electrical stimulator to activate during the stance phase after adjusting for a delay such that the stimulus occurred post‐heel strike but prior to mid‐stance. This instance was chosen because the H‐reflex depicts phase‐dependent modulation with facilitated response around the mid‐stance (Chalmers & Knutzen, 2000; Johannsson et al., 2019). The stimulation intensity was increased until the M‐wave's p‐p amplitude plateaued (M_max_, Figure 2b). Gaussian and sigmoid functions were fitted to obtain p‐p amplitudes of the H‐reflex and M‐wave, respectively. Each function was weighted, with higher weights for the most relevant 30% of the H‐reflex, to best fit the curve over the region of interest (i.e., H_max_) and thus most accurately determine the max value of the H‐reflex. Stimuli were applied in randomized intervals of 9–16 s to avoid post‐activation depression (Pierrot‐Deseilligny & Mazevet, 2000), avoiding habituation to the timing and preventing saturation or under‐recruitment of motor neurons.

##### Standardization of H‐reflex stimulation intensity

In order to ensure that stimulations during the subsequent conditions conformed to the ascending slope of the recruitment curve, the stimulus intensity was standardized at 70% of the H_max_ determined from the participant's respective recruitment curve Figure 2b). This intensity was chosen as it is known to elicit a submaximal but sufficiently large reflex that is reproducible and sensitive to changes in synaptic efficacy without being confounded by the saturation effects present at higher intensities (Bouguetoch et al., 2020; Crone et al., 1990; Pinniger et al., 2001). In addition, the inclusion criterion for the H‐reflex data was predicated on the preceding M‐wave amplitude being within the limits established by the range, set as percentage range relative to the baseline M‐wave amplitude (e.g., within ±20% of the mean M‐wave amplitude for each condition). We are aware that consistent inclusion is crucial as it serves as a reliable indicator of consistent H‐reflex. This ensures that variability in H‐reflex amplitudes can be attributed to our experimental manipulations rather than inconsistencies in stimulus application (Knikou, 2008). The resulting H‐reflexes were recorded and then normalized to the background EMG activity to control for variations in muscle activation levels, thus ensuring consistency in our reflex measurements.

#### 2.3.2 3‐D kinematics

The 3‐D trajectories of all markers on both feet (heel, base metatarsus three, first and second metatarsus heads) were captured at a sampling frequency of 500 Hz and used to extract the footfall kinematics. Kinematics were low‐pass filtered (Butterworth, zero‐phase lag, fourth‐order, 25 Hz cut‐off frequency) before identifying heel strike and toe‐off events using a foot velocity algorithm (O'Connor et al., 2007). Two consecutive heel strikes of the same leg were defined as a stride. Stride time was calculated for both feet independently as the time elapsed between two consecutive ipsilateral heel strikes, and stride length was determined as the Euclidean normalized vector on foot coordinates at toe‐off and its consecutive heel strike. Step width and length were calculated in the medial–lateral (ML) and anterior–posterior (AP) direction, respectively, as the distance between the line of progression of two consecutive ipsilateral heel strikes and the position of the contralateral heel marker at its heel strike. Finally, the mean and variability of footfall parameters was evaluated as the inter‐stride mean and SD of the parameters, respectively, for each participant in each condition. This involves calculating the mean and the SD for the sequence of strides made by each participant. The resulting values measure the overall adjustment in footfall placement and the consistency of these adjustments from stride to stride.

##### Extrapolated centre of mass and margins of stability

**FIGURE 4:**
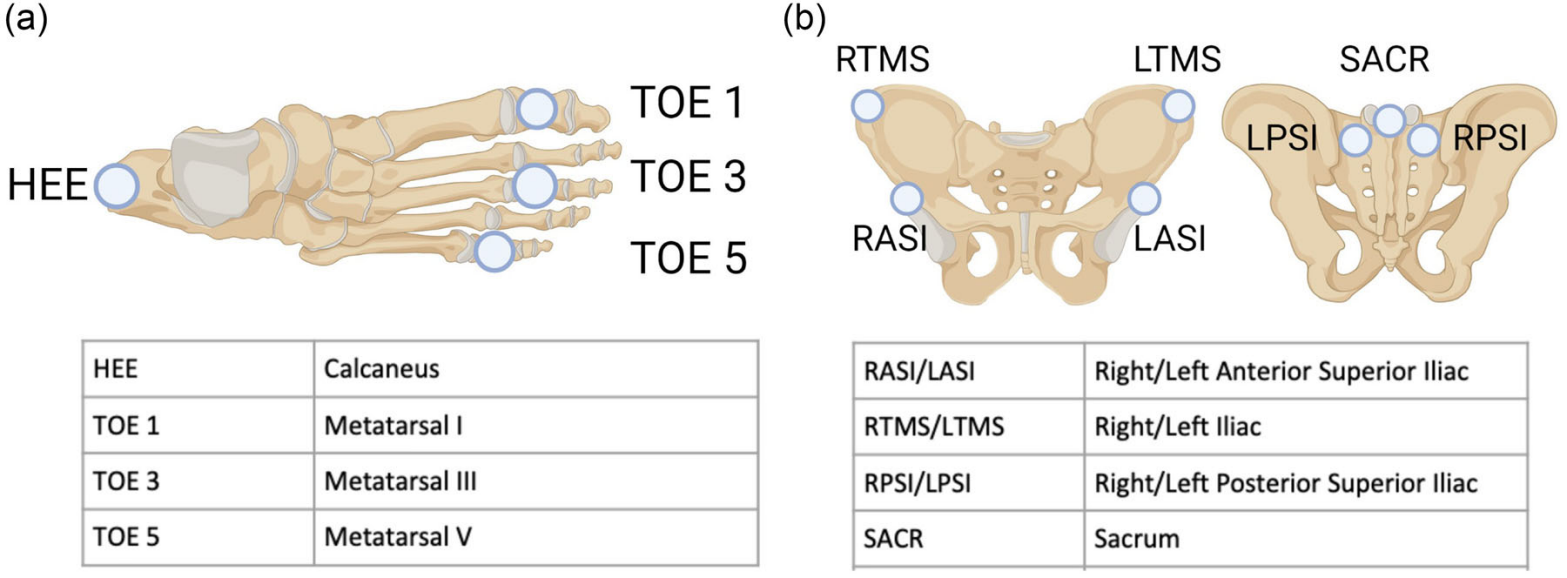
(a) Three markers were placed on the foot, defined by the first and fifth metatarsal markers (TOE 1, TOE 3, TOE 5). (b) Seven markers were placed on the pelvis, and the centroid was used to estimate the CoM of the whole body.

Extrapolated centre of mass (XCoM) is a dynamic representation of the body's CoM that considers both the position and velocity of the CoM (Figure 3). Margin of stability (MoS) is then evaluated (Hof et al., 2005) as the distance between the body's XCoM and the boundaries of the BoS (the area covered by the feet, Figure 4a). MoS provides a dynamic measure of the body's stability at any given point.

The position of of body's CoM ($\mathrm{Co}M_{\mathrm{position}}$) was approximated from the centroid of the seven pelvis marker trajectories (L/RASI, L/RTMS, SACR, L/RPSI—Figure 4b); followed by evaluating the velocity of CoM (CoM_velocity_) from the first derivate of its position. The XCoM was derived as follows and calculated at heel strike (Hof, 2008; Hof et al., 2005; Hof et al., 2007):

(1) $$\mathrm{XCoM}=\mathrm{CoMp}+\frac{\mathrm{CoMv}}{\sqrt{\frac{g}{l}}}$$

where 𝑙 is the pendulum length (leg length), illustrated as the inverted pendulum model (Winter, 1995), defined as the distance from the CoM to the midpoint between the two malleoli markers of the foot at heel strike, and 𝑔 is the gravitational constant defined as *g* = 9.81 m/s^2^ (Figure 3a).

The MoS was computed in both the AP and ML directions, and was obtained by evaluating the distance between the XCoM and BoS

(2) MoS=BoS−XCoM

LTO5 and RTO5, representing the fifth metatarsal marker of the left and the right foot, were used as the lateral boundary for the BoS. LTO1 and RTO1 were the anterior boundary (Figure 3b).

#### 2.3.3 Statistical analyses

##### Effect of weight conditions on H‐reflex and background EMG

A linear mixed model ANOVA with weight conditions, bEMG and participants as independent variables and H‐reflex amplitude the dependent variable was conducted to assess the effect of varying loading conditions on H‐reflex response. A separate analysis was conducted to test whether weight conditions significantly affected bEMG.

##### Relationship between H‐reflex, background EMG and lower body kinematics

Linear mixed model ANOVAs were conducted to investigate the effect of weight conditions as independent variables on lower body kinematics (inter‐stride means and SD in footfall kinematics, and MoS) as dependent variables. In addition, separate linear mixed model ANOVAs were conducted in order to investigate the association between normalized H‐reflex (H‐reflex_bEMG_), H‐reflex, bEMG and weight conditions were added as independent variables and lower body kinematics as dependent variable.

For all analyses, participants were included as a random effect and weight conditions as a fixed effect (five levels: Normg, Wei_20_, Wei_40_, Har_20_, Har_40_) factors. The significance level was set at 5%. Tukey's *post hoc* approach was used to investigate the changes in the dependent variables between the different weight conditions compared to the baseline—Normg. The statistical analysis was performed in R (version 4.1.3, R Core Team, Vienna, Austria).

## 3 RESULTS

### 3.1 Background EMG and H‐reflex

The impact of weight conditions on the H‐reflex and bEMG_stance_ was significant (*P* < 0.05; Table 1, highlighted in grey). A linear increase in the H‐reflex and bEMG_stance_ was observed, ranging from the extreme unloading condition (Har_40_) to additional loading (Wei_40_), with an approximate 46% and 52% increase, respectively, achieving statistical significance among these extreme test conditions. Detailed *post hoc* comparisons revealed that bEMG_stance_ activity under the Har_20_ and Har_40_ conditions was not significantly different in comparison to the baseline, Normg (*P* > 0.05). Conversely, in the weighted conditions, Wei_40_ displayed a statistically significant increase in bEMG_stance_ (42% increase, *P* < 0.05; and a non‐significant ∼19% increase in Wei_20_) compared to Normg, underscoring the influence of additional weight on muscle activation.

**TABLE 1:** Mean (SD) and variability (SD) of footfall, CoM, H‐reflex and bEMG for each condition.

**Mean gait parameters per condition**	**Condition**	**Stride time (s)**	**Stance time (s)**	**Swing time (s)**	**DLS (s)**	**Stride length (cm)**	**Step length (cm)**	**Step width (cm)**
	Har_40_	1.24 (0.12)	0.75 (0.07)	0.47 (0.04)**	0.13 (0.02)**	131.81 (13.63)	66.45 (6.77)	**7.22 (3.1)†**
	Har_20_	1.21 (0.08)	0.76 (0.05)	0.45 (0.02)	0.15 (0.02)**	131.32 (10.84)	66.1 (5.43)	7.82 (2.09)
	Normg	1.21 (0.07)	0.79 (0.07)	0.43 (0.03)	0.18 (0.02)	132.07 (10.29)	67.27 (4.3)	8.1 (2.43)
	Wei_20_	1.19 (0.08)	0.78 (0.06)	0.41 (0.02)	0.18 (0.02)	132.04 (6.69)	66.65 (3.32)	8.5 (2.23)
	Wei_40_	1.21 (0.07)	0.81 (0.07)	0.42 (0.03)	0.19 (0.03)	133.65 (10.93)	67.51 (5.5)	**8.85 (3.25)†**
**Gait variability per condition**	Har_40_	0.03 (0.01)***	0.02 (0)	0.02 (0.01)***	0.01 (0)	4.25 (0.99)**	2.96 (0.59)**	1.27 (0.31)***
	Har_20_	0.02 (0)	0.02 (0)	0.01 (0)	0.01 (0)	3.51 (0.54)	2.61 (0.44)	1.43 (0.23)
	Normg	0.02 (0)	0.02 (0)	0.01 (0)	0.01 (0)	3.38 (0.64)	2.38 (0.38)	1.87 (0.45)
	Wei_20_	0.02 (0.01)	0.02 (0)	0.01 (0)	0.01 (0)	3.41 (0.43)	2.49 (0.37)	2.08 (0.48)
	Wei_40_	0.02 (0.01)*	0.02 (0.01)	0.01 (0)***	0.01 (0)	4 (1.1)	2.81 (0.59)*	2.31 (0.55)**
**Mean per condition in margin of stability (MoS)**	**Condition**	**MoS_ML (mm)**	**MoS_AP (mm)**	**Variability in margin of stability (MoS)**	**MoS_ML (mm)**	**MoS_AP (mm)**		
	Har_40_	90.38 (11.35)	352.07 (32.99)***		13.92 (2.99)**	17.86 (2.98)**		
	Har_20_	89.73 (9.05)	372.24 (24.6)**		12.98 (2.43)**	18.38 (4.34)***		
	Normg	93.85 (12.85)	399.86 (20.57)		10.41 (1.94)	13.8 (1.81)		
	Wei_20_	98.83 (12.57)	408.35 (24.83)		11.02 (1.4)	14.16 (1.53)		
	Wei_40_	98.82 (9.61)	414.93 (30.23)		12.23 (2.06)*	17.05 (3.3)*		
**Mean bEMG_swing_ and non‐nomalized H‐reflex per condition**	**Condition**	**bEMG_swing_ (mV)**	**H‐reflex (mV)**	**Mean bEMG_stance_ and normalized H‐reflex per condition**	**bEMG_stance_ (mV)**	**H‐reflex_bEMG_**		
	Har_40_	0.03 (0.01)	**0.58 (0.27)†**		0.11 (0.05)	6.48 (3.71)		
	Har_20_	0.04 (0.03)	0.62 (0.31)		0.13 (0.06)	5.55 (2.48)		
	Normg	0.04 (0.04)	0.63 (0.31)		0.12 (0.05)	6.11 (5.37)		
	Wei_20_	0.04 (0.05)	0.77 (0.43)		0.14 (0.06)	6.09 (3.3)		
	Wei_40_	0.04 (0.02)	**0.87 (0.4)†**		0.17 (0.06)***†	5.39 (2.04)		

*Note*: The mean values show the average response, while the variability is calculated as the inter‐stride standard deviation. Grey cells denote significant main effects of weight conditions (*P* < 0.05). Pairwise *post hoc* comparison tests highlight significant differences from the baseline—Normg (*P < 0.05; **P < 0.01; ***P < 0.001) and between extreme weight conditions (†*P* < 0.001).

Notably, no significant association was observed between the weight conditions and variability in both bEMG_stance_ and the H‐reflex. Additionally, there was no significant association between bEMG_stance_ and the H‐reflex, suggesting that the H‐reflex is not the result of changes in bEMG_stance_. Furthermore, we found no association between weight conditions and the normalized H‐reflex (H‐reflex_bEMG_), indicating that changes in loading did not significantly impact these normalized values.

### 3.2 Relationship between H‐reflex and footfall (spatio‐temporal gait parameters) kinematics

Seven footfall kinematics were investigated: stride time, stance phase, swing phase, double limb support time (DLS), stride length, step width and step length. No differences (*P* > 0.05) were found for any parameter between the two legs, and therefore analysis from only the dominant leg is reported. The results of linear mixed effect model ANOVA revealed a significant association between the predictor variable weight conditions and the outcome measures of mean swing time, DLS and step width (*P* < 0.05, Table 1, in grey). Table 1 also presents *post hoc* analyses, showing significant changes in mean swing time (Har_40_) and DLS across weight‐reduced conditions (Har_20_ and Har_40_) in comparison to Normg (*P* < 0.01; Table 1, values in bold).

ANOVA on outcome measures of variability demonstrated a significant effect of weight conditions on almost all parameters of gait (*P* < 0.05 for variability in stride and step length; *P* < 0.01 for stride time and step width, *P* < 0.001 for swing phase; Table 1, grey cells), except stance phase and DLS. *Post hoc* analyses presented significantly altered variability during extreme weight conditions (Har_40_ and Wei_40_) for all parameters except stride length, where only Wei_40_ led to significantly different behaviour compared to Normg. The H‐reflex presented a significant effect on the stance and swing phase (*P *< 0.05), but H‐reflex_bEMG_ showed no association with any of the mean and variability of gait parameters.

### 3.3 Relationship between H‐reflex and MoS

The weight conditions significantly affected mean MoS_AP (*P* < 0.05), and *post hoc* analysis demonstrated significant differences only in the weight reduction conditions (Har_20_ and Har_40_ *_,_ P *< 0.01).

The weight conditions significantly affected the variability of MoS in both AP and ML directions and *post hoc* analysis revealed significant differences in only the unloaded (Har_20_ and Har_40_ *P* < 0.01; Table 1, in bold) vs the Normg condition. Contrastingly, in ML inter‐stride variability of MoS was significantly different in unloaded (Har_20_ and Har_40_ *P* < 0.01; Table 1, in bold) as well as the extreme additionally loaded (Wei_40_ *, P* < 0.05; Table 1, in bold) vs the Normg condition.

The H‐reflex and H‐reflex_bEMG_ showed a significant association only with mean MoS_AP (*P *< 0.05), and otherwise H‐reflex_bEMG_ showed no association with any other MoS parameters. Finally, the interactive effects of weight conditions and H‐reflex were not observed on any gait parameters (footfall or CoM kinematics).

## 4 DISCUSSION

Using the H‐reflex, this study investigated the understanding of human locomotion, particularly in how gait and motor control dynamically adapt to various weight‐bearing conditions, a fundamental aspect of daily activities. We considered the normal condition as a baseline for the changes observed in other conditions. Our findings highlight the challenges posed by weight reduction conditions as demonstrated by a shorter DLS (25%), a longer swing time (by 9%), a narrower step width (by 11%), and notably increased stride and swing time variability (48% and 74%, respectively) in relation to normal walking. More notably within extreme conditions, our study observed a 46% increase in the H‐reflex in the Wei_40_ condition compared to the Har_40_ condition, alongside a 47% lower swing time variability. However, the non‐significance of relationships between the H‐reflex and gait variability suggests the complexity of the neural control mechanisms governing adaptations in locomotion.

### 4.1 Relationship between weight conditions and excitability of neural pathways

Weight conditions significantly influenced bEMG_stance_ and the H‐reflex, with an overall increase of 52% and 46% from Har_40_ to Wei_40_ conditions, respectively. A similar observation was seen where there was an increase in the H‐reflex during weight‐added conditions during standing (Tsuruike et al., 2012), while a decrease in H‐reflex was seen during standing and walking under various anti‐gravity conditions: 1 g – 0.25 g (Ferris et al., 2001; Tsuruike et al., 2012). Changes in H‐reflex response due to weight addition may be induced from reduced inhibition, requiring higher lower‐limb muscle activation and stiffness (Ferris et al., 2001; Tsuruike et al., 2012). This change is especially prominent in the soleus muscle, generating nearly all body support during the mass‐bearing phase (Tsuruike et al., 2012), possibly leading to a higher H‐reflex response. Furthermore, this outcome is intuitive because humans are accustomed to handling additional loads, thus allowing more ‘short latency or feedforward’ mechanisms to manage task demands. In contrast, the reduced H‐reflex response observed under weight reduction is probably due to reduced soleus activation during the stance phase of gait, emphasizing a shift in regulatory balance control mechanisms. As the transition to weight reduction is less common in our daily lives, one likely shift could be heightened reliance on supra‐spinal contribution for negotiating increases in task demands (Shadmehr & Krakauer, 2008; Wolpert et al., 2011). This suggests a fascinating adaptability of our nervous system, where the interplay between supra‐spinal and spinal level control is modulated based on the task demands and environmental context (Kim et al., 2015).

Interestingly (non‐normalized) H‐reflex magnitude saw an 8% decrease versus a corresponding 38% increase for the extreme weight conditions compared to Normg (*P* < 0.05). After normalizing the H‐reflex (H‐reflex_bEMG_), these trends changed, from an increase by 6% to an ∼11% decrease in magnitude for 40% weight reduction versus addition conditions, respectively, compared to the baseline condition (*P* > 0.05). In literature, H‐reflex normalized to bEMG_stance_ is often regarded as gain of the afferent pathway within the spinal cord circuitry (Pierrot‐Deseilligny & Mazevet, 2000; Zehr, 2002). However, due to a disproportionately high increase in bEMG_stance_ under the extreme weight addition condition, we observe a reversal in trends in association across the two opposing extreme weight conditions. The results on normalization therefore should be interpreted with caution as they might indicate an underestimation of the impact of the H‐reflex on task performance.

### 4.2 Relationship between weight conditions, excitability of neural pathways and footfall kinematics

The results demonstrated a significant association between weight conditions and the mean swing time, DLS and step width outcome measures. No significant effects of weight conditions on stride time and stride and step length are possible because AP movement should align with the treadmill speed—set at each individual's preferred walking speed.

The unloaded conditions (Har_20_ and Har_40_) showed significant changes in mean swing time and DLS compared to Normg, which has also been observed in another study (Barela et al., 2019; Dragunas & Gordon, 2016; König Ignasiak et al., 2019). Findings for the extreme weight‐reduced condition (Har_40_) reveal interesting behavioural adaptation—a shorter DLS with a longer swing time and narrower step width with a significantly greater stride and swing time variability—compared to the baseline condition. A shorter period of DLS is associated with a larger gait variability (Brach et al., 2005; Hausdorff, 2005; Owings & Grabiner, 2004). Furthermore, narrowing step width allows a natural approach for better control, low energy cost and reduced risk of injury, and is often seen in patients with Parkinson's disease (Damm et al., 2020; Hausdorff et al., 1998; Konig, Singh, et al., 2016). Despite issues on the interpretability of MoS in the AP direction as a measure of stability (Bruijn et al., 2013; Hof et al., 2007), we did notice a 12% decrease in MoS_AP compared to Normg. Taken together, unloading at 40% body weight led to a narrower step width, a narrow MoS in the AP direction and considerable increases in temporal variability. These changes would suggest unloading imposed restrictions on adaptability as well as the ability to negotiate balance during walking.

Contrary to the unloading results, no substantial effect of weight‐added conditions was observed on the mean of all gait parameters, except a 10% increase in step width from Normg to the extreme weight‐added condition (Wei_40_). In contrast, all gait parameters except for the stance phase, stride length and DLS saw significant modifications in variability with weight addition, albeit only under the Wei_40_ condition (notably around 26% increase in step width variability). Alteration in step width, as observed in our study, is one of the most common strategies to compromise the perturbation (Hof et al., 2007); placing the feet wider produces a broader BoS, creating a higher safety margin and preventing an overshoot of the CoM over the outside BoS border. Wider step width generated a larger mean MoS_ML, ensuring a more comprehensive platform for the CoM and XCoM to ensure stability (Hof, 2008; McAndrew Young et al., 2012; Watson et al., 2021; Yang & King, 2016). However, minor changes in other parameters suggest that the movement of CoM and XCoM was largely unaffected, meaning overall stability can be maintained solely by foot placement. A concurrent increase in step width variability implies that this wider foot placement must be continually regulated to accommodate task demands from additional loading.

Regarding its role in lower body kinematics, the H‐reflex demonstrated an association with mean MoS_AP and variability in the timing of both stance and swing phases, signifying its effect on influencing gait phase timings and overall stability. The fact that H‐reflex magnitude correlates with stance time variability is not surprising as we stimulated after heel strike and before mid‐stance to account for phase‐dependent facilitation of the H‐reflex (Chalmers & Knutzen, 2000; Ferris et al., 2001; Johannsson et al., 2019). This correlation is also reflected in our finding of higher H‐reflex under additional weight‐loading conditions compared to Normg. Furthermore, the association of H‐reflex magnitude with swing phase variability suggests a potential role of SOL excitability in the planning of subsequent foot placement. Swing time variability was higher for both Wei_40_ and Har_40_ than baseline, but the variability value was lower for Wei_40_ than Har_40_, with H‐reflex linearly reducing with weight reduction. Upon normalization of H‐reflex with bEMG_stance_, this relationship was only observed for mean MoS_AP, indicating the role of the excitatory status of the muscle on the planning of foot placement during walking.

Behaviourally, our participants found Har_40_ more challenging to negotiate than the weight addition gait task. This is unsurprising as they were all young, asymptomatic adults familiar with carrying backpacks. In contrast, weight reduction conditions are more common in rehabilitating neurological patients. Physiologically, by assessing the capability to activate motor neurons, given other conditions such as presynaptic inhibition and phase‐dependent modulation remain unchanged, the H‐reflex assesses the nervous system's response under varying task demands (Palmieri et al., 2004). While we did observe associations between the H‐reflex response and gait performance, we cannot conclusively state whether H‐reflex excitability led to or reflects (or a combination of the two) the alterations in the timing of foot placement and overall stability. However (although further research is warranted), a strong relationship between H‐reflex amplitude and variability in the timing of foot placement observed in our study suggests that the mean value of the H‐reflex might provide feedback for the sensorimotor system that the task at hand requires greater and finer regulation (Thompson et al., 2019; Thompson & Wolpaw, 2021). The physiological implications suggest that both monitoring and modulating motor neuron excitability in the lower extremity musculature should be a relevant target for interventions to improve gait stability and reduce the risk of falls in various populations (Phadke et al., 2007; Thompson et al., 2009, 2019; Thompson & Wolpaw, 2014, 2015; Thompson & Wolpaw, 2021), such as older adults or individuals with neurological disorders.

The insights gained will deepen our understanding of fundamental locomotor strategies in healthy individuals and provide valuable benchmarks for assessing and treating gait abnormalities. Thus, our research stands to make a significant contribution to the field of gait rehabilitation, offering potential implications for developing more effective rehabilitation treatment. These individualized treatment protocols cater to the specific challenges and needs of diverse patient populations.

### 4.3 Limitations versus scope

Treadmill walking does not accurately reflect over‐ground walking; step width is more pronounced, and variability is minor during treadmill walking, but it is insensitive to MoS at heel strike (Rosenblatt & Grabiner, 2010). In addition, gait characteristics are mainly less variable in the direction of movement (Hollman et al., 2016), potentially due to mechanical constraints and a more cautious gait (Yang & King, 2016). This can certainly be seen in marginal modifications that were observed in mean and variability of stride time and length in our study. Furthermore, the body weight system provides passive stabilization but is not dynamic in correspondence to the movement, restricting the movement of the CoM in all directions (Dragunas & Gordon, 2016). Despite these issues, both harness systems and treadmills continue to be used in clinics (Hidler & Wall, 2005; Knikou et al., 2013; Pennycott et al., 2011) to support body weight in patients suffering from severe injuries for rehabilitation during movement tasks.

### 4.4 Conclusion

Our study investigated a critical aspect of human locomotion—the ability to adjust gait and motor control dynamically in response to the diverse mechanical loads encountered in everyday life. Such loads range from the simplicity of walking unburdened to the complexity of manoeuvring with additional weight, such as carrying groceries or wearing a backpack. By focusing on how these common yet variable weight‐bearing conditions influence gait, our study aimed to uncover the intricate processes by which the nervous system orchestrates these adaptive responses. Our findings particularly highlight the challenges posed by unfamiliar weight‐reducing conditions and the capabilities of our nervous system in negotiating acute balance adaptations, demonstrated by a 25% shorter DLS, a 9% longer swing time, an 11% narrower step width, and notably higher stride and swing time variability (48% and 74%, respectively), compared to the baseline condition. Furthermore, the association of the H‐reflex with stance and swing time variability suggests that increasing task demands led to modifications in motor neuron excitability and likely served as feedback to the nervous system for enhancing the regulation of foot placement and overall stability. Future research may further explore the specific nature of this relationship between the H‐reflex and the timing of foot placement in other cohorts, such as older adults or other pathological groups, to rigorously test the relationship and provide clues for the rehabilitation of these population groups.

## AUTHOR CONTRIBUTIONS

Conceptualization: Navrag B. Singh, Heiner Baur and Yong Kuk Kim. Methodology: Yong Kuk Kim and Navrag B. Singh. Investigation: Yong Kuk Kim and Navrag B. Singh. Formal analysis: Yong Kuk Kim and Navrag B. Singh. Writing—original draft preparation: Yong Kuk Kim. Writing—review and editing: Yong Kuk Kim, Michelle Gwerder, Heiner Baur, William R. Taylor and Navrag B. Singh. Supervision: Navrag B. Singh and William R. Taylor. All authors have read and approved the final version of this manuscript and agree to be accountable for all aspects of the work in ensuring that questions related to the accuracy or integrity of any part of the work are appropriately investigated and resolved. All persons designated as authors qualify for authorship, and all those who qualify are listed.

## CONFLICT OF INTEREST

The authors declare no conflicts of interest.

## FUNDING INFORMATION

No funding was received for this study.

## Data Availability

The data that support the findings of this study are available on request from the corresponding author. The data are not publicly available due to privacy or ethical restrictions.
